# Mandibular Symmetrical Bilateral Canine-Lateral Incisors Transposition: Its Early Diagnosis and Treatment Considerations

**DOI:** 10.1155/2016/5043801

**Published:** 2016-12-29

**Authors:** Yehoshua Shapira, Tamar Finkelstein, Rana Kadry, Shirley Schonberger, Nir Shpack

**Affiliations:** Department of Orthodontics, The Maurice and Gabriela Goldschleger School of Dental Medicine, Tel Aviv University, Tel Aviv, Israel

## Abstract

Bilateral mandibular tooth transposition is a relatively rare dental anomaly caused by distal migration of the mandibular lateral incisors and can be detected in the early mixed dentition by radiographic examination. Early diagnosis and interceptive intervention may reduce the risk of possible transposition between the mandibular canine and lateral incisor. This report illustrates the orthodontic management of bilateral mandibular canine-lateral incisor transposition. Correct positioning of the affected teeth was achieved on the left side while teeth on the right side were aligned in their transposed position. It demonstrates the outcome of good alignment of the teeth in the dental arch.

## 1. Introduction

A tooth may deviate from its normal path of eruption usually as a result of severe crowding or presence of an obstacle such as a supernumerary tooth or an odontoma. Such eruption deviation can occur with no apparent local or systemic cause, resulting in ectopic eruption of the tooth in a place normally occupied by another permanent tooth. The most frequently ectopically erupted tooth is the mandibular permanent lateral incisor which may occur unilaterally and bilaterally [1–4]. A study on the occurrence of ectopic erupting permanent teeth has shown that 30% involved the mandibular permanent lateral incisors unilaterally and bilaterally [5].

Early diagnosis of a disturbed eruption of a mandibular permanent lateral incisor can be made in young children during the early mixed dentition at the age of 6–8 years, though some variation in timing of eruption of that tooth has been reported [6]. The permanent lateral incisor during this period is in its preeruptive migration and the deciduous lateral incisor root is resorbing. The lateral incisor may, for unknown reasons, deviate from its normal eruption path and become distally displaced, resulting in overretention of the deciduous lateral incisor, and could ectopically erupt in a transposed position with the permanent canine [7].

Tooth transposition is defined as an interchange in position of two adjacent permanent teeth in the same quadrant of the dental arch or eruption of a tooth in a place normally occupied by another tooth [8]. It is a type of ectopic eruption that results in an abnormal sequence of the permanent teeth in the dental arch. Transposition occurs most frequently between the maxillary canine and first premolar and occasionally between the maxillary canine and lateral incisor [9, 10]. Rare cases of transposition between a canine and a second premolar or an incisor have been reported [11]. Transposition of a tooth may be complete, where both the crowns and roots of the involved teeth are in transposed position. It may be incomplete when only the crown is transposed but the root is within its normal place. Unilateral transpositions are more often than bilateral ones, with left side predominance, and are found more often in females than in males [11, 12].

Transposition in the mandible is relatively rare and occurs between the canine and lateral incisor and is usually unilateral. Only few cases of bilateral transposition of a canine and lateral incisor in the mandible have been reported [13, 14]. The prevalence of tooth transposition varies according to different studies and was found to be 0.43% of patients in India [15], 0.38% in Turkish population [16], and 0.14% of patients in Nigeria [17, 18], whereas the prevalence of mandibular canine-lateral incisor transposition is only 0.03% [19].

The etiology of transposition is unknown and the reason why a tooth deviates from its normal path of eruption is still obscure. Several theories have been suggested such as genetic factors [20, 21], interchange in the position of the developing tooth buds, early loss or prolonged retention of deciduous teeth, and trauma and mechanical interference to the erupting permanent teeth [11, 21].

Tooth transposition has been reported to be associated with other dental anomalies such as missing teeth, small or peg-shaped maxillary lateral incisors, retained deciduous mandibular lateral incisors and canines, rotations and malposition of adjacent teeth, and root dilacerations and impactions [22].

The literature on early detection and treatment procedures for this abnormality is relatively sparse. The purpose of this article is primarily to emphasize early diagnosis and detection of bilateral mandibular tooth transposition and describe its orthodontic management and outcome.

## 2. Clinical Diagnosis and Evaluation

The early mixed dentition period, between 6 and 8 years, is the best time for assessing the development and path of eruption of the mandibular permanent lateral incisors. These age group children are usually first examined by a pediatric or general dentist who should evaluate both the dental health condition and the dental development. Using a panoramic radiograph is very useful for early diagnosis of the position and path of eruption of the unerupted teeth.

## 3. Clinical and Radiographic Examination

**Figure 1:**
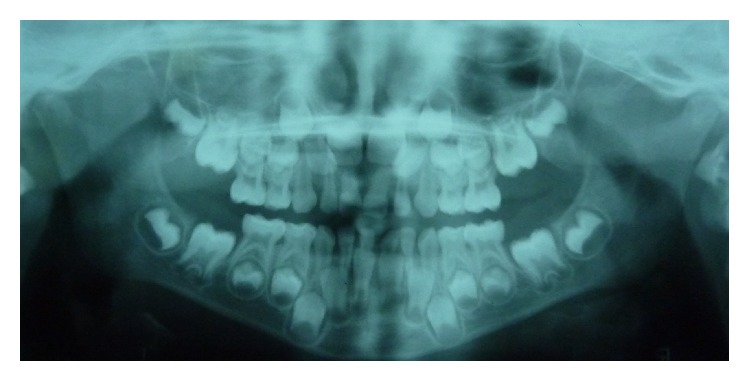
Panoramic radiograph of a 6-year-old boy with normal dental position and development of the mandibular permanent lateral incisors.

**Figure 2:**
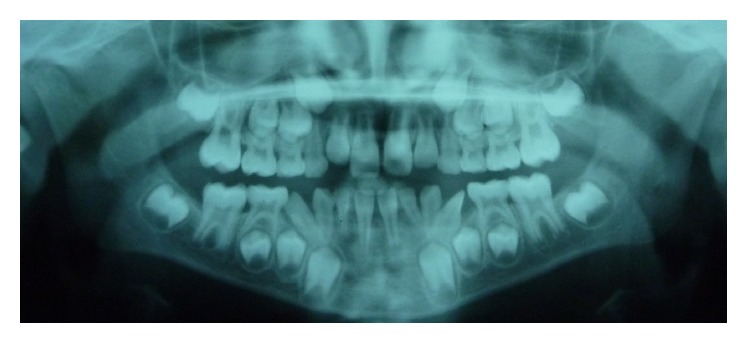
Panoramic radiograph at the age of 8 years, showing the mandibular permanent lateral incisors ectopically erupted and rotated in the place of the first deciduous molars causing their early exfoliation.

**Figure 3:**
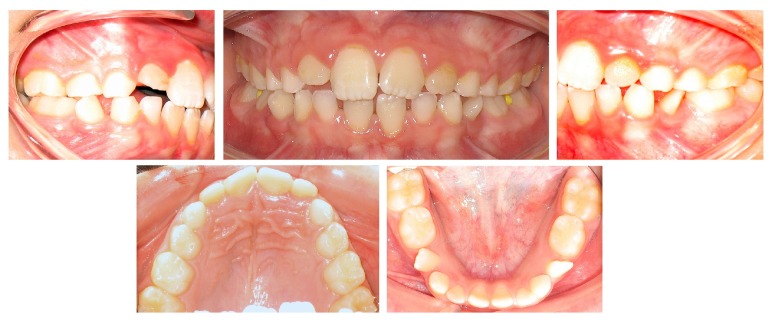
Pretreatment intraoral photographs showing the mandibular right and left lateral incisors ectopically erupted rotated in the place of the early exfoliated first deciduous molars.

A routine panoramic radiograph of a 6-year-old boy, taken at the Pedodontic Department of Tel Aviv University School of Dental Medicine, demonstrated normal dental position and development of the mandibular permanent lateral incisors, which are expected to erupt into their proper position in the arch uneventfully (Figure 1). Surprisingly enough and for unknown reason, a follow-up panoramic radiograph taken two years later, at the age of 8 years, demonstrated bilateral distal deflection of the mandibular permanent lateral incisors bypassing the deciduous lateral incisors and canines and ectopically erupted rotated in the place of the deciduous first molars causing their early exfoliation (Figure 2). His intraoral examination revealed Class I interarch relationship with normal overbite and overjet in the early mixed dentition. The mandibular permanent lateral incisors have ectopically erupted bilaterally distal to the deciduous canines with 90 degrees of rotation, causing early exfoliation of the deciduous first molars (Figure 3).

## 4. Treatment Objectives

The primary objectives were to derotate the mandibular permanent lateral incisors and upright and reposition them to their normal position next to the central incisors. This will allow the canines and first premolars to erupt into their normal place and avoid the possible development of transposition between the canines and lateral incisors.

## 5. Treatment Plan, Procedure, and Outcome

**Figure 4:**
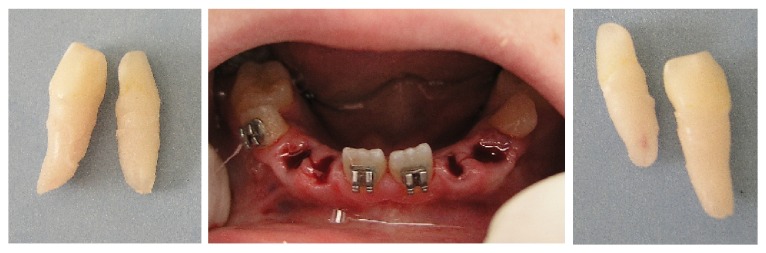
Extraction of the deciduous lateral incisors and canines.

**Figure 5:**
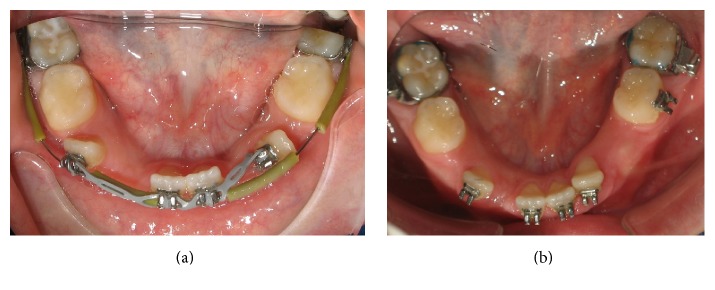
Derotation (a) and mesial movement of the permanent lateral incisors (b).

The early diagnosis is of crucial importance for establishing a correct treatment planning. The retained deciduous lateral incisors and canines were immediately removed at the age of 8 years (Figure 4). Edgewise fixed appliances were used, first to correct the severely rotated lateral incisors and upright them and then to move them mesially to their normal place next to the central incisors (Figure 5).

**Figure 6:**
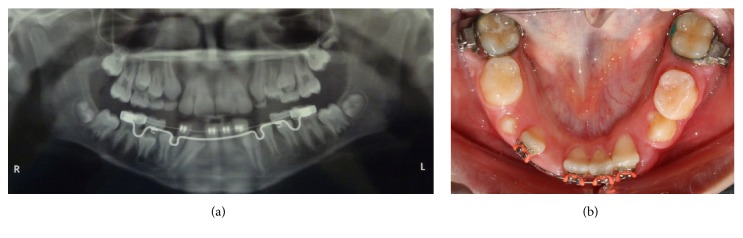
Panoramic radiograph (a) and intraoral photograph (b) showing the right permanent canine in position to erupt between the lateral and central incisors.

**Figure 7:**
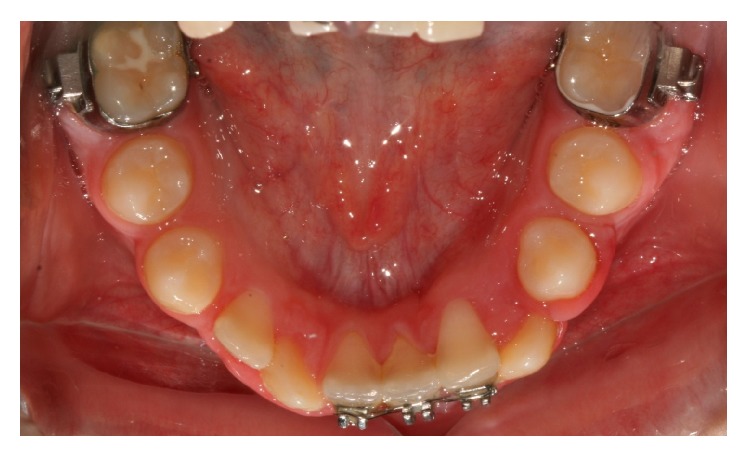
The right permanent canine is erupting in transposition with the lateral incisor. The left permanent canine is erupting into its normal position in the arch.

**Figure 8:**
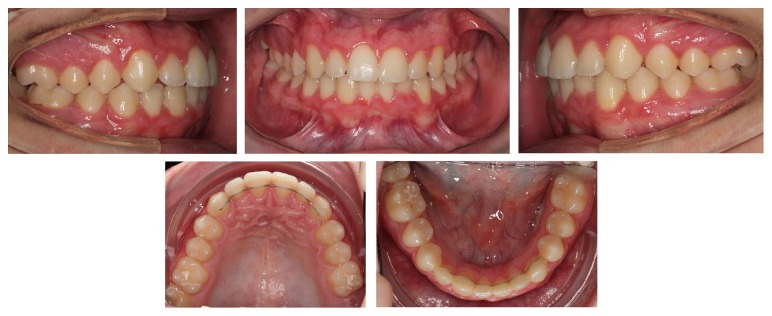
Posttreatment intraoral photographs showing mandibular right canine in complete transposition with the lateral incisor, while the left canine is in its normal position in the arch.

**Figure 9:**
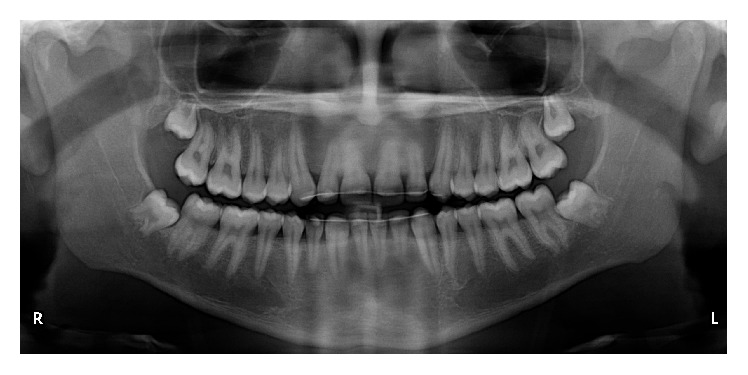
Posttreatment panoramic radiograph showing complete transposition between the mandibular right canine and lateral incisor. The left canine erupted in its normal position.

Periodic radiographs taken during treatment showed that the right permanent canine was already erupting between the central and lateral incisors, while the left canine and lateral incisor were almost overlapping each other (Figure 6). It would have been dangerous to continue the movement of the right lateral incisor to its normal position as it could cause interference between their roots and possible root resorption. Therefore, it was decided at that point that it would be safer to align them in their transposed position. The left lateral incisor was uprighted and moved to its normal place next to the central incisor allowing the left canine to erupt into its normal position in the arch, while the right permanent canine was erupting in transposition with the lateral incisor (Figure 7). The right canine's cusp tip was slightly reshaped to resemble an incisor. Following completion of the orthodontic treatment at the age of 12 years permanent retainers were bonded on the upper and lower anterior teeth. The very nice outcome of the treatment is presented in the final intraoral photographs (Figure 8) and panoramic radiograph (Figure 9).

## 6. Discussion

The developing mandibular permanent lateral incisor normally resorbs the root of the deciduous tooth during the process of eruption into the oral cavity. It is still unclear what causes a tooth to deviate from its normal path of eruption and erupt ectopically. The presence of an obstacle such as a supernumerary tooth or an odontoma could be a factor causing the deflection and migration of a tooth. Several theories have been suggested as etiological factors to explain why a tooth deviates from its normal path of eruption to become transposed: interchange in position of the anlage at the very early stage of tooth development [23], genetic control within the dental follicle [20, 21, 24], prolonged retention of the deciduous lateral incisor [25, 26], crowding and inadequate arch length [2]. Crowding did not seem to be a primary cause of the anomaly as sufficient space to accommodate all the permanent teeth was found in the presented case, which is in agreement with previous reported cases [11]. Another possible explanation suggested that the retained mandibular deciduous lateral incisor could be the cause of the displacement of the permanent lateral incisor resulting in transposition [27].

It is not yet clear whether the retained deciduous tooth is the cause or the result of the displacement and ectopic eruption of its successor.

Treatment considerations for transposed teeth include repositioning them in their normal place in the dental arch, maintaining them in their transposed position, or extracting one of the transposed teeth.

In managing treatment for mandibular tooth transposition several factors should be considered such as the amount of distally displaced lateral incisor and the intrabony position of the permanent canine. Early detection of the abnormal eruption path of the lateral incisor allows for early intervention by uprighting and moving the lateral incisor to its normal place in the arch prior to the eruption of the canine into transposition with the lateral incisor. This was successfully achieved in our presented case only on the left side. On the contralateral side, however, the position of the canine was already between the central and lateral incisors and to avoid a possible risk of root resorption it was allowed to erupt into complete transposition with the lateral incisor. The canine's cusp tip was reshaped to resemble a lateral incisor.

## 7. Conclusions

Early detection of a distally displaced mandibular permanent lateral incisor at the early mixed dentition, at the age of 6–8 years, and timely interceptive intervention may reduce the risk of tooth transposition in the mandible and avoid complex orthodontic therapy. The early orthodontic management and treatment outcome of mandibular bilateral canine-lateral incisor transposition have been described.
